# Dr. Wattmann on the Entrance of Air into the Veins, &c.

**Published:** 1847-04

**Authors:** 


ART. X.

*Sicheres Heilverfahren bei dem Schnell gefährlichen Lufteintritt in die Venen und dessen gerichtsärztliche Wichtigkeit.* Von Dr. CH. JOS. EDL. v. WATTMANN, k. k. n. ö. Regierungsrathe, Leibchirurg, o. ö. Professor der praktischen Chirurgie und der ersten chirurgischen Klinik, Vorsteher der Operations-Institutes an der k. k. Universität in Wien, &c.—*Wien,* 1843.

*A Certain Remedy in rapidly dangerous Entrance of Air into the Veins, and on the Medico-legal Importance of that Event.* By Dr. CH. JOS. VON WATTMANN, Councillor to the Imperial Government and Surgeon to the Emperor of Austria, Professor of Practical and Clinical Surgery, and Director of the Institute for Operations in the Imperial University of Vienna, &c.—*Vienna,* 1843. 8vo, pp. 188.

THE subject both of the artificial introduction and of the spontaneous, or rather accidental, entrance of air into the veins has been several times considered in this Journal, especially in the Number for October 1838, pp. 455 et seq. Dr. Wattmann's publication again brings this very interesting question before us, so far as regards the spontaneous or accidental admission of air into the veins of man. It might indeed have been expected that little room for discussion would remain after the numerous experiments, and the nearly as numerous disputations, both oral and written, which the subject has called forth; and it certainly is to be regretted that a phenomenon of such unquestionable importance, and of which so many alleged examples have occurred in the practice of surgery, should still be a matter of doubt and dispute.* As an example of how unsettled opinions are respecting it, we may mention that in four systems of surgery, now in course of publication in Paris, three different doctrines are advanced; M. Nelaton admits, M. Ph. Boyer denies, that the spontaneous entrance of air into the human veins has ever proved fatal; while MM. Bérard and Denonvilliers take a middle course between these extremes, and think "that the solution of the problem remains doubtful, and is not decided either affirmatively or negatively." In this last opinion M. Vidal de Cassis coincides, in the second edition of his work on Surgery, which has just issued from the press. We must, however, observe that M. Vidal, the most recent of the writers just mentioned, has scarcely devoted sufficient attention to the subject. He states that MM. Bérard and Denonvilliers have collected almost all the recorded cases purporting to be examples of spontaneous entrance of air into the human veins during an operation: the fact being that they have not collected one fourth of them. He thinks it a remarkable and happy circumstance, that no new cases of the accident have been heard of since the publication of M. Bouillaud's Report, and the academic discussion thereon (examined in this Journal, Oct. 1838), and thence insinuates an argument against the reality of the event. A glance at the table of cases we have drawn up will show how much M. Vidal is mistaken in this respect. He states that the influence of exhaustion from hemorrhage in accelerating the fatal consequences of air admitted into the veins of dogs has been established by M. Gerdy particularly. M. Gerdy has,

* Since the above lines were written, we have seen that one of the questions to be submitted to the Medical Section of the Scientific Congress of France, at its meeting at Marseilles, 1st September, 1846, is as follows: "Fixer autant que possible nos idées sur l'influence de l'introduction de l'air dans les veines pendant les opérations."

on the contrary, endeavoured to show that the alleged effect of exhaustion is at the least doubtful. M. Vidal further implies that surgeons have invented spontaneous entrance of air into the veins, as an excuse for the sudden death of their patients during the performance of operations, inasmuch as no one, he says, has ever spoken of its occurring except consequent on wounds inflicted by a surgeon. In this also M. Vidal is utterly mistaken; the presence of air in the veins has been observed after decapitation, and also in some cases of suicide, and death has in some of these latter cases been attributed to its influence; again Sir C. Bell relates (Practical Essays, 1841, p. 11), that Baron Larrey, on looking over his Sketches of the Wounded at the Battle of Waterloo, "fixed with interest on the case of a young man who had been wounded in the lower part of the neck. Well, I know, said this excellent surgeon, how that man must have died. I have seen so many wounded during my campaigns, and die from air drawn into the veins." We fully admit that there has been a great deal of exaggeration respecting the entrance of air into the veins; like many other things, it had a run not merely with the profession, but even became for a time, we might almost say, a fashionable source of apprehension out of the profession. The wife of an eminent orator and leading member of the French Chamber of Deputies was bled at the arm, and having been enjoined to observe the strictest quietude, was left alone in her bedroom: after a short time hemorrhage came on, and, in the absence of timely help, the lady lost a quantity of blood sufficient in her condition to prove fatal. The catastrophe was, however, attributed to entrance of air into the veins, and this explanation attaining due publicity in the newspapers of the day, produced such an impression, that for some time, in certain circles, few physicians cared to propose venesection, and still fewer patients to submit to the operation. But though we agree with Blandin, Velpeau, and many others, that sudden death during operations has been erroneously attributed in several cases to entrance of air into the veins, we shall examine whether there are not other recorded cases which establish both the reality and fatality of the phenomenon. It may seem, from the title of Dr. Wattmann's book, that we are about to travel beyond the limits of his inquiries, but such is not the fact, for though he purports only to consider how the effects of the admission of air into the veins is to be remedied, and the medico-legal import of such an occurrence, he, in point of fact, enters into a consideration of the whole subject as a question of physiological surgery.

As we have to deal solely with *spontaneous* entrance of air into the veins, we shall refer but little, and that only incidentally, to the experiments on injecting air into the venous system. But we may say thus much, that many of the results and theories of modern experimenters were anticipated by some two hundred years. Not only did Woepfer, R. J. Camerarius, Redi, Bohn, Ant. Heydin, Brunner, Harderus, Valisneri, and Sproegelius kill animals by injecting air into their veins, but Camerarius, Brunner, and Valisneri ascertained that a certain quantity of air might be thus injected with impunity. Valisneri also discovered that the effects of air thrown into the veins varied in different animals, as dogs were more easily killed by it than .sheep. The diversity of symptoms produced, did not escape notice. Brunner observed opisthotonos, Sproegelius slight convulsions, while Camerarius never witnessed either rigidity or spasms of the muscles, but, on the contrary, found them relaxed. Camerarius and

Harderus ascertained by dissection the presence of air in both the arteries and the veins, and Sproegelius thought the blood was always preternaturally liquid. Of the theories advanced in late years to explain how air in the organs of circulation causes death, three at least were anticipated. Brunner, Camerarius, Harderus, and Sproegelius attributed death to suspension of the circulation, from the heart being so distended that its contraction is prevented, and Harderus further held the opinion, that the fibres of the heart were weakened or paralysed by extreme distension, just as is the case with the urinary bladder in certain cases of retention of urine. Morgagni too agreed that death resulted from disturbance of the functions of the heart. Finally, Bohn conceived that air proved fatal by acting as a powerful and rapid poison. (Morgagni De Sed. et Caus. Morb. Epist. v. sect. 21 et seq.)

The discovery of the spontaneous entrance of air into a wounded vein, is comparatively, indeed absolutely, of recent origin. It is true Mery observed that if the abdominal cava of a living animal is punctured, the vein becomes filled with air as it empties itself of blood, but he thought the air was contained in the radicle veins, and flowed from them towards the cava, and thence to the heart. (Mém. de l'Acad. des Sc. 1707, p. 167.) Littré not long after found air in the blood-vessels after death from hemorrhage. (Ibid. 1714, p. 330.) Redi and Caldesi had seen air circulating with the blood in some cold-blooded animals, as vipers and tortoises, and thought it existed naturally in their blood-vessels, but Haller pointed out that air was not present, unless some vessel had been opened. Nysten remarks that he several times saw the veins and right auricle of persons who had been decapitated distended with air (Recherches de Physiolog. et de Chim. Patholog. 1811, p. 5), but failed like his predecessors to surmise its real origin. Verrier, a veterinary surgeon, seems to have been the first who, in 1806, recognized the spontaneous entrance of air into the veins of an animal. Magendie subsequently (Journal de Physiolog. 1821) inferred the occurrence of the event in man from M. Beauchene's case, and sought to establish it by experiments on animals; but as he allowed the air to enter through a tube passed into a vein, its admission in those experiments cannot be termed spontaneous. Subsequent to Magendie's first researches, several cases were observed in which air was supposed to have accidentally entered the human veins, but those cases did not attract very much attention at the time, and their interpretation was and still is disputed. M. Amussat, we believe, was the first who actually demonstrated the spontaneous passage of air into an open vein. His first communication on the subject is a short and incidental, but very explicit, passage in his memoir on traumatic hemorrhage, read to the Academy of Medicine in 1835, and published in their Memoirs, t. v, pp. 68 et seq. For his subsequent researches we refer to the Number of this Journal already mentioned, and to his 'Recherches sur l'Introduction accidentelle de l'Air dans les Veines,' &c.

We would now willingly proceed to examine the alleged cases of this accident in man, but as their import must be tested by comparing how far the circumstances attending them agree with the causes which produce entrance of air into the veins, and with the phenomena observed in animals that have undoubtedly died from its effects, we must first advert to those causes and phenomena, the more especially as considerable difference of opinion and statement exist respecting both.

The experiments of Barry, modified and confirmed by those of M. Poiseuille, fully establish the influence of atmospheric pressure on the venous circulation. There is no question that during inspiration, the flow of venous blood towards the thorax is facilitated in the great venous trunks in the vicinity of that cavity, while, during expiration, a partial reflux occurs in the same vessels. It is further generally assumed, from the experiments of Poiseuille, that the suction thus exerted on the contents of the veins extends to but a very short distance from the superior aperture of the thorax, because the veins being flexible tubes,—with very delicate yielding parietes, incapable of contracting in the direction of their length,—communicating with a cavity which tends to exhaust them by suction,—and exposed to atmospheric pressure external to that cavity, the suction cannot act beyond certain limits. For just as when we attempt to empty with a syringe a flexible tube full of liquid, and fixed at both ends, a small quantity of liquid only enters the syringe, because atmospheric pressure forces the sides of the emptied portion of the tube into contact, so do the veins when similarly circumstanced collapse. The diastole of the right ventricle of the heart is admitted by some to exert an influence on the venous circulation, quite similar in kind to, but less in amount than that of the expansion of the thorax during inspiration. MM. Gerdy, Velpeau, and others, indeed deny this influence of the heart, because opening one side of the thorax has been found to immediately arrest the entrance of air into an open vein, but we think that its reality is established beyond a doubt. In M. Bouillaud's Report, already referred to, it is expressly stated that the flux and reflux of blood was sometimes observed to be synchronous with the action of the heart, and in some experiments the characteristic sound of air entering the veins coincided for a time with inspiration, but then became more rapid, and was obviously isochronous with the action of the heart. M. Barthélemy independently made similar observations, (Bull. de l'Acad. t. i, p. 904, and t. ii, p. 368); and Dr. Wattmann, in one case in man, distinctly observed the venous pulse in the internal jugular vein coincide with the action of the heart. (p. 114.) But whatever may be the influence exerted by the action of the right cavities of the heart, the aspiration of the venous blood does not extend beyond certain limits, and M. Poiseuille having found that the external jugular vein of a dog when laid bare collapsed to within 4 centimeters (1½ inch) of the thorax during inspiration, this was thought to give an approximate measurement of the sphere of the phenomenon. Wherever then this suction, manifested by the venous pulse, exists, it is admitted that air may and frequently does enter a vein when wounded; and as the obstacle to the entrance of air into a vein beyond the limits of the venous pulse, is the obliteration of the canal of the vein by atmospheric pressure, the sphere within which air can enter a vein may obviously be extended if the collapse of the vein is prevented. As regards a considerable extent of the internal jugular vein, the whole length of the subclavian vein and the upper half of the axillary vein, M. Bérard has shown (Archiv. gen. t. xxiii, pp. 169-71) that they are united to the adjacent bones and muscles by fibrous attachments, which prevent their collapsing under atmospheric pressure, and consequently they are liable to admit air when opened. Again, the committee appointed to report on M. Amussat's experiments, found that air entered the axillary vein beyond the limits of the venous pulse when the parietes of the vein were kept tense and the wound of the vein was kept open, and it is admitted that the limits of aspiration may be extended if the collapse of a vein is prevented by any cause,—such as disease of its coats,—adhesion to a diseased structure,—or if traction is exerted on it during an operation, as when, for example, a tumour in which a vein is involved is put on the stretch to facilitate its dissection. How far the limits of aspiration may be extended by those or similar causes, has not been precisely determined. MM. Velpeau, Gerdy, Blandin, and others, deny that it can be propagated very far, or to such veins as the external jugular, the mammary, or the subscapular; while M. Amussat and many others maintain that it may extend to the branches of the internal jugular, subclavian, and axillary veins. This is a point of great importance; for, according to our conclusion respecting it, many of the alleged cases of entrance of air into the veins must be absolutely rejected on the one hand, or admitted to be at least possible on the other.

It is obvious that, unless something counteracts the effects of atmospheric pressure, superficial veins, such as the external jugular, must collapse when suction is exerted on them, and that they cannot consequently either exhibit the venous pulse, or admit the aspiration of air through them, unless atmospheric pressure is obviated. But M. Barthélemy (loc. cit.) has several times observed distinct undulations, isochronous with the action of the heart, extend to a considerable height in the external jugular vein, which filled from below upwards, and collapsed from above downwards, and he has also seen air enter the vein when venesection was performed on it in those cases. M. Pellis also distinctly saw a flux and reflux of blood in the external jugular vein of the human subject, in a case referred to in the table inserted further on. But M. Barthélemy farther found that air may enter the external jugular vein when no such undulations exist, and at a point higher up than they ever extend to. Thus in one horse, air entered the external jugular vein, opened above its lower third as for ordinary venesection, three times in 2½ minutes, and produced the usual symptoms, and in a similar experiment on a second horse, air entered freely without any blood flowing externally; air likewise readily entered a canula, (not as in Magendie's experiments, and those mentioned in Bouillaud's Report passed down a vein to the region of the venous pulse, but) simply inserted into an opening in the upper third of the jugular vein; and finally, M. Barthélemy found air, without the aid of a canula, enter the external jugular vein immediately below the facial vein in sufficient quantity to cause death. M. Boulley and several others have also seen air enter the external jugular vein of the horse during venesection, but the foregoing facts sufficiently demonstrate that air may enter a vein far beyond the limits assigned by M. Velpeau, or even by M. Amussat, and that its admission into the external jugular vein, even at its upper extremity, is not constantly prevented by atmospheric pressure. Sir Charles Bell has, we think, offered the true explanation of this fact. During inspiration, the platysma myoides, the sterno-cleido mastoid, and the anterior portion of the trapezoid muscles, are, he says, "lifted off the veins of the neck, and, by removing the atmospheric pressure from them, allow them to dilate" (Op. cit. p. 17); and the same effect is produced by exertion, by raising the shoulder, and by certain motions of the head and chest. Sir C. Bell, it is true, seems to confine this explanation to the internal jugular and subclavian veins, but it also applies to the other veins of the neck, to the external jugular, for example, which lies beneath the platysma myoides muscle. The action of the muscles of the neck then, during a sigh, a deep inspiration, a constrained posture, or a sudden motion of the head or shoulder, may render the entrance of air into the external jugular vein possible, and it will be seen that in one case in man (that recorded by M. Pellis), the alleged or, as we think, actual admission of air into this vein occurred at the moment the patient threw his head back and inspired deeply. This consideration also explains the difficulty with which air entered the veins in the experiments recorded in M. Bouillaud's Report; for in those experiments the veins were laid bare to a considerable extent, and the obstacle afforded by the muscles to the action of atmospheric pressure was consequently removed. A circumstance dwelt on by Sir Charles Bell must not be overlooked. We shall quote his words.

"When water flows through a tube, the tube being gradually larger at its further extremity, and a lesser tube inserted into it, water will not flow from the larger tube into the smaller, but from the smaller into the larger. This corresponds with the course of the blood in the veins; for the lesser veins are inserted into a series of trunks gradually enlarging in their diameters, till they reach the heart. In these circumstances, a hole in the side of the tube will not discharge water, but will admit air." (Op. cit. p. 15.)

This hydraulic law will not of course apply to the veins, as they are not rigid tubes, unless they are prevented from collapsing under atmospheric pressure by some of the influences already adverted to. Another circumstance which Dr. Wattmann particularly notices may greatly favour the entrance of air into veins which lie above the horizontal level of the heart; the veins so situated are seldom completely full, they rarely convey as much blood as they are capable of containing, and when a vein so circumstanced is opened, and its parietes are at the same time prevented from collapsing, air must readily enter the vessel only partly filled with blood. This is the reason why the vertical posture favours the entrance of air into the veins, and the influence of previous hemorrhage in favouring the phenomenon, or at least in hastening death, may probably, partly at least, arise from the admission of air being facilitated by the diminution of the quantity of blood in the venous system.—The conditions necessary for the occurrence of the admission of air into small veins lying beyond the ordinary limits of aspiration may not frequently exist, but this does not invalidate their reality. The experiments of M. Barthélemy, and the observations of MM. Verrier, Boulley, Gerard, and Leblanc (referred to in the table we have constructed), establish, we think, the fact of the spontaneous entrance of air into the external jugular vein; that fact, we also think, is explained by the considerations just adverted to; and the same explanation applies to other small veins of the neck.

The signs and *symptoms* of spontaneous entrance of air into the veins as observed in animals, are generally as follows. A noise, variable in kind and intensity, is heard at the orifice of the vein, from which either immediately or after some time frothy blood sometimes issues, and a bruit de souffle with or without gargouillement is heard, on auscultation, in the right cavities of the heart. There is agitation, with indications of anxiety, the breathing becomes accelerated and laboured, and the pulse frequent. From this condition the animal often recovers, but if the symptoms become aggravated, the respiration and pulse get weak, the animal trembles, totters, and falls, the urine and fæces are passed involuntarily, the limbs are stiffened, or convulsed, or affected with tetanic spasms, and the breathing and pulse cease to be perceptible. The animal may still gradually revive from a state of apparent death, or death may occur within a period varying from perhaps a minute to some hours. Of those symptoms some are constant, viz. the acceleration and embarrassment of breathing and frequency of the pulse, and also, in aggravated cases, suspension of respiration and of the action of the heart, with insensibility. But almost all, if not all, the other symptoms are liable to variations which it is necessary to notice.

The noise caused by air entering a vein is compared in dogs to the sound made by those animals in lapping, and in horses to a gurgle (*glou-glou, gargouillement*). But this sound may be absent, thus, in the 7th experiment contained in M. Bouillaud's Report, no sound was at first heard after the subclavian vein had been opened, but air had nevertheless entered the vein as appeared from the embarrassed and accelerated respiration, and also from the more positive evidence of bubbles of air mixed with blood issuing from the vein. Again, in the 5th experiment, a "*lapement*" was heard at first, but after a time ceased, though large bubbles of air continued to enter the wound, and in the 12th experiment, on the contrary, the air entered noiselessly at first, but afterwards with its appropriate sound. The sound seems to be most commonly and most distinctly heard during inspiration, but in the 1st and 2d experiments of the commission, so often referred to, the entrance and exit of air, at first synchronous with inspiration and expiration, subsequently corresponded to the pulsations of the heart; again, though the sound is usually intermittent, it may be continuous, at least nothing is said of its intermitting in the 8th, 9th, and 10th experiments, and we may directly infer that it was continuous in the 11th, as we are told that it was louder during inspiration. In experiments 1 and 2, no urine or fæces were evacuated. In experiments 1, 2, 6, 11, 32, and 33, there were no convulsions. Sometimes the animals uttered cries, sometimes they did not. The rapidity of death in the fatal cases, also varied greatly. The most rapid death occurred in three minutes, in the 11th experiment, in which the animal, a dog, was held in the vertical posture. It is to be observed that none of the experiments of the commission on animals in the erect position were fairly conducted, except the foregoing one; in all the others, the dogs were placed in the horizontal posture once or oftener during the course of the experiment, and in some the orifice of the vein was also closed. Thus, in the 14th experiment, apparent death supervened, *almost immediately* when the animal was placed upright, but on replacing it horizontally and closing the orifice in the vein, it revived until again placed vertically. In many instances, too, the access of air was interrupted by the formation of clots on the opening in the vein, and obstruction from this cause, occasioned by the greater coagulability of the blood of dogs, seems to be one reason why dogs often resist the action of air better than men. But if we go beyond the experiments of the commission, we shall find that death may occur in the dog as rapidly as in any of the alleged cases in man; for M. Segallas states that in one experiment, which he performed on the jugular vein of a dog, death was almost instantaneous. (Bull. de l'Acad. t. ii, p. 427.) The period within which death occurs varies moreover, there is some reason to believe, in different animals, Valisneri states that dogs die more rapidly than sheep, and the experiments in M. Bouillaud's Report indicate that death occurs in horses not sooner, but in a much less variable period than in dogs. The erect position undoubtedly hastens death, the rapidity with which the air enters, has of course an immense influence, as have also, though in a much less degree, the conformation and state of health of the individual, for M. Barthélemy found that horses with a small thorax or any affection of the lungs are easily killed by injecting air into the veins. (Bull. de l'Acad., t. ii, pp. 370-5.)

As regards the post-mortem appearances after spontaneous entrance of air into the veins, M. Bouillaud states that the right cavities of the heart were, in the experiments on which he reported, *constantly* distended with air; air was found in the left cavities of the heart of one dog only. In horses, on the contrary, air was found not only in the right heart, but also almost constantly in the left cavities of the heart, in the aorta, and other arteries, and in the vessels of the brain; a difference which M. Bouillaud thinks probably arises from the ultimate ramifications of the pulmonary vessels being larger in horses than in dogs, and consequently more readily permitting the frothy blood to pass to the left side of the heart. In three dogs killed, after 4, 4½, and 8 days respectively, air was found in the right cavities of the heart and in the arteries of the second animal, and in the arteries of the third animal, but no air was any where discovered in the first animal, whose heart, especially the right cavities, was remarkably flaccid.

Even if this account accurately applied to every case, it follows that the post-mortem appearances present some variety. The presence of air in the left side of the heart and in the arteries of the horse, evidently does not arise from those animals living longer after admission of air into the vascular system than dogs do, for it was found in those situations in the 35th, 36th, 31st, and 37th experiments, in which the horses respectively survived but 12½, 13, 14, and 16 minutes, which was not the case with dogs, that lived upwards of an hour; when, however, dogs survive a considerable time we have already seen that the air makes its way to the arterial system. But the appearances after death are by no means constantly such as are above described, and it is not unimportant to notice some of the occasional discrepancies. M. Bouillaud, we have seen, describes the presence of air in the right cavities of the heart as a *constant* phenomenon when death occurs soon after entrance of air into the veins, and M. Amussat further maintains that this air is never free, but is always mixed into a froth with the blood, which has assumed a bright arterial colour; this, it is said, is so constantly the case, that we may thereby determine whether air entered the vascular system during life or after death, in the former case the appearances are those just mentioned, but if the air is free and unmixed with blood, it gained admission after death. This distinction is said to be important in a medico-legal point of view, and also in determining the real cause of death in some of the alleged fatal cases of entrance of air into the veins during an operation. (Bull. de l'Acad. t. ii, p. 277.) Now in the first place, the heart does not constantly contain air after death from its admission into the veins. M. Poiseuille in some instances, found the right cavities of the heart contain blood only, without a particle of air. (Gaz. Méd. de Paris, 1837, p. 728, noté.) Neither is the air, when present, always mixed with blood; in the 7th experiment mentioned in M. Bouillaud's Report, "*pure*" air and liquid blood were found unmixed in the right auricle, and in the 6th experiment there was free air unmixed with blood in both the right cavities of the heart. Again, when air and blood are mixed, they do not always occur as froth, in some instances liquid blood preponderates. (Exp. 15.) In others (Exp. 16) the blood forms a coagulum with bubbles of air disseminated through it, a condition which we shall find existed in one case in the human subject.

Before we compare any of the recorded cases in the human subject with the symptoms and appearances observed in animals, it is convenient to give a tabular view of all the alleged cases of the kind which we have been able to collect; and we have added to this table several cases in which air has entered the external jugular vein of the horse accidentally during venesection. In order to economise space, we have occasionally thrown matter under the head of observations which should rather be found in another column. Some of the cases which we have inserted should, perhaps, have been omitted. Thus the three first cases are certainly not examples of death from air entering the veins: in Klein's case there is nothing to justify the presumption that death arose from this cause; as to the second case, Dr. Busse (Gaz. Méd. de Paris, 1838, p. 114) informs us that at the time of its occurrence, it was mooted as an hypothesis whether death might not have been the result of air entering the veins, but that dissection showed this conjecture was unfounded. Mr. Barlow's case is not recorded by that gentleman as an example of the accident in question, though Velpeau refers to it as if it was. The three next cases are quite unauthentic. As regards the first of them (4th in the table), Dr. Lodge wrote a very improbable and vague account of it from Paris, to Dr. Warren in America; no one knew anything of it in Paris, its authenticity has been often impugned, and never, so far as we know, vindicated by Dr. Lodge: indeed it seems scarcely possible to credit the alleged circumstances of the case. Dupuytren, we are told, when about to divide an enlarged saphena vein, expressed some apprehension lest air might enter the vessel, he nevertheless divided it, the peculiar sound of the admission of air was heard, and the patient fell dead; now besides that, M. Poiseuille has shown that the pressure of the blood in the femoral vein is greater than that of the atmosphere, whence entrance of air into a vein of the lower extremity is impossible, it is incredible that Dupuytren, if apprehensive that air might enter the vessel, should have failed to compress it above the point at which he divided it. The fifth case is also valueless; all we know of it is that Dr. Warren, in Hays's 'American Cyclopædia,' &c., says that Dr. Stevens "witnessed" death from air entering a vein during an operation, and again in his surgical observations on tumours, states that such an event occurred "within Dr. Stevens's knowledge." We know not from what source the report of the case said to have occurred in Sir A. Cooper's practice originally emanated, nor have we anywhere found it mentioned, save as a rumour, respecting which no particulars are given, and for which no authority is cited; it is thus M. Ollivier, to whom we refer in the table, mentions it. We have nevertheless tabulated all those cases, because they have been all referred to more or less frequently as alleged examples of the accident in question. Many of the other cases are also more or less doubtful, and several of them, we think, are quite inconclusive, but to this we shall presently return.

| CASES. | REFERENCE. | REGION OR VEIN. | SYMPTOMS AND RESULT. | DISSECTION. | OBSERVATIONS. |
|---|---|---|---|---|---|
| 1. Klein, 1814. | Graefe and Walter's Journal, 1820, pp. 120-6. | Extirpation of thyroid gland. | Death in less than a minute. | No dissection. | |
| 2. Graefe, 1822. | Gazette Méd. de Paris, 1839, p. 261. | Amputation of breast; gland in axilla. | Syncope, and death during removal of gland in axilla. | No important organ wounded. Heart and great vessels natural. | |
| 3. Barlow, 1830. | Med.-Chir. Trans., 1830, vol. xvi, p. 19. | Varicose vein on cheek. | Copious hemorrhage. Syncope. Recovery. | | |
| 4. Dupuytren (Lodge and Warren). | American Journ. of Med. Sci., Aug. 1832; Gaz. Méd., 1833, p. 227. | Internal saphena vein. | Peculiar noise. Rapid death. | No dissection. | |
| 5. Stevens (Warren). | Hay's Amer. Cyclopædia of Med. and Surg., vol. i, p. 263. | | | | |
| 6. Sir A. Cooper (Ollivier). | Dict. de Méd., 2d ed., t. ii, p. 70. | | | | |
| 7. Schuster, 1837. | Gaz. des Hôp., 1843, p. 33. | Median basilic vein (venesection). | Flow of blood suddenly ceased. A hissing sound. Arm grasped above and frothy blood expressed from the puncture. No general symptoms. | | Bandage had been so tight as to impede arterial circulation. On loosening it blood flowed again as usual. |
| 8. Simon, 1841. | Ann. de la Chirurg. Fr. et Etrang., 1842. p. 380. | Median basilic vein (venesection). | A *snuffling* noise; a bubble of air seen at puncture. Insensibility, spasmodic respiration. Puncture closed. Cold aspersions. Recovery in a minute and a half. | | The blood ceasing to flow, the bandage had been loosened and a second puncture made. When patient revived the bandage was found quite loose. |
| 9. Mott, 1828. | Amer. Journ. Med. Sci., Nov. 1828, p. 127; Med.-Chirurg. Trans., vol. xvi, p. 32. | Facial vein. | Gurgling noise. Embarrassed breathing. Irregular violent action of the heart, face distorted, general convulsions. Hemiplegia. Apparently impending death. Recovery in about half an hour. | | The hemiplegia continued half the day. |
| 10. Amussat, 1837. | Bull. de l'Acad. de Méd., 1837, t. i, p. 894. | Amputation of breast; vein below clavicle. | A whizzing sound. Patient expressed fear of dying, and became faint. Pressure on wound. Repeated pressure on sternum. Recovery. | | |

| | | | | | |
|---|---|---|---|---|---|
| 11. Duval (Toulmouche). | Bull. de l'Acad., 1837-8, t. ii, p. 146. | Amputation of breast. | Sound like a prolonged inspiration. Syncope. Apparent death. Recovery. | | The arm was abducted when the symptoms occurred. |
| 12. Clemot, 1830. | Gaz. des Hôp., 1830, t. iv, No. 24, p. 95. | Small vein, supposed to be external jugular (tying subcl. artery). | Sound of aspiration ceasing and recurring as finger was placed on or removed from vein. No general symptoms. Vein tied. Recovery. | | |
| 13. Pierre and Cadougnes, 1835 (Wattmann). | Sicheres Heilverfahren, &c., p. 11. | External jugular vein (venesection). | Sluggish flow of blood. Sudden whistling sound. No general symptoms. Vein immediately closed. Recovery. | | |
| 14. Rigaud, 1836. | Quelques Faits de Pratique Chirurg., p. 166. | External jugular (tying subcl. artery). | Sound of aspiration repeated three times. No general symptoms. Recovery. | | |
| 15. Malgaigne, 1836. | Gaz. Méd., 1836, p. 167. | External jugular vein. | Distinct gurgling sound. No general symptoms. Both ends of vein tied. Recovery. | | |
| 16. Koeple. | Provinc. Med. and Surg. Journ., 1842, p. 200. | External jugular vein. | A hissing sound. Patient uttered a cry and *instantly* fell senseless. Immediate compression on vein. Ligature round base of tumour. Recovery in ten minutes. | | |
| 17. Warren, 1830. | Surgical Obs. on Tumours, p. 298. | Vein near carotid artery. | Bubbling sound. Bubbles of air seen in wound. Face livid. Pulse slow. Stertorous breathing. Convulsive spasms. Vein compressed. Temporal artery opened. Recovery. | | |
| 18. Delaporte, 1836. | Bull. de l'Acad., t. i, p. 13[illegible]. | Tumour on right side of neck. | Distinct hissing sound. Gurgling in thorax. Patient exclaimed she was dying. Syncope. Recovery. | | The mouth was drawn to one side, and numbness felt in both hands, which continued in the right hand a month after. |
| 19. Mayor, 1838. | Bull. de l'Acad., 1839, t. iii, p. 934. | Ditto. | *Bruit de ronflement.* Immediate insensibility. Apparent death. Instant pressure on wound. Repeated compression on lower third of sternum. Recovery in a few minutes. | | The tumour passed under the posterior border of the sternomastoid muscle, which was divided. The symptoms came on when the deep attachment of the tumour was being separated. |

| CASES. | REFERENCE. | REGION OR VEIN. | SYMPTOMS AND RESULT. | DISSECTION. | OBSERVATIONS. |
|---|---|---|---|---|---|
| 20. Wattmann, 1825. | Sicheres Heilverfahren, &c., pp. 7 and 119-20. | Branch of axillary vein. | Whistling sound. Immediate syncope. Pressure on wound. Axillary vein tied. Recovery. | | The extremity continued cold and numb till the collateral circulation was established. |
| 21. Ditto, 1826. | Ditto. | Ditto. | Ditto. | | Ditto. |
| 22. Clemot. | Gaz. des Hôp., 1830, t. iv, No. 24, p. 95. | Ditto. | Sound of aspiration. Syncope. Vein through which air entered tied. Recovery. | | The symptoms were so sudden and alarming, that the assistants fled and left M. Clemot alone with the patient. |
| 23. Capellati. | Provinc. Med. and Surg. Journal, 1842, p. 200. | Axilla. | A hissing noise. Patient exclaimed she was dying. Pulse irregular. Breathing laborious. *Apparent* insensibility. Compression on wound. Usual remedies in syncope. Recovery in ten minutes. | | The patient remained conscious the whole time, but was unable to speak, and |
| 24. B. Cooper, 1843. | Med.-Chir. Trans., 1844, vol. xxvii, p. 41. | Amputation at shoulder joint. | Gurgling sound. Instant collapse, threatening immediate death. Pulse fluttering. Respiration quick. Feeble sighing. Urine and fæces passed involuntarily. Wound closed. Usual remedies in syncope. Horizontal posture. Recovery. | | An hour elapsed before the patient could be removed to bed with safety. |
| 25. Warren. | Surg. Observ. on Tumours, p. 182. | Internal jugular vein. | A few bubbles of air entered open mouth of vein, but were arrested and forced back by finger. No general symptoms. Recovery. | | The vein was intentionally divided and the finger previously placed on it below. |
| 26. Wattmann, 1823. | Sicheres Heilverfahren, &c., pp. 5 and 110. | Ditto, *right* side. | Hissing sound. Immediate syncope. Recovery on placing finger on vein, on removing, recurrence of the sound and of the syncope. Dull, rustling sound heard in thorax. Horizontal posture. Cold aspersion. Orifice in vein pinched up in forceps, and ligature tied round it. Gradual recovery. | | The orifice in the vein was distinctly seen. No blood flowed from it, and the flux and reflux in it corresponded to action of heart. The hissing sound ceased on the lips of the orifice in the vein being separated. Both the *left* extremities were paralysed for some days. |
| 27. Erichsen. | Edinb. Med. and Surg. Journal, No. 158, p. 19. | Internal jugular vein. | Sound of air entering vein. No other symptom. Recovery. | | Sound heard when vein was raised to tie it below a wound inflicted in attempt at suicide. |

| | | | | | |
|---|---|---|---|---|---|
| 28. Velpeau, 1837. | Bull. de l'Acad., 1837, t. i, p. 896; Gaz. Méd., 1838, p. 116. | Internal jugular vein. | A hissing sound, followed by bubbling in the wound. Patient exclaimed she was dying and fainted. Compression deep in wound. Usual remedies in syncope. Recovery. | | Velpeau does not state what vein was opened in his first account of this case: in his second report he says the internal jugular vein was wounded. |
| 29. Begin, 1837. | Presse Médicale, 1837, p. 463. | Ditto. | A gurgling sound. No bad consequences. | | |
| 30. Asmus, 1842. | Br. and For. Med. Rev., Oct. 1842, p. 556. | Ditto. | A bubbling sound. Patient exclaimed all was over. Convulsions, alternating with opisthotonos. Syncope. Bubbling repeated. Pressure on vein. Stimulants. Recovery in about 12 minutes. | | The moment after the bubbling was heard, the patient said he was well. There was *no venous hem* till after the bubbling was heard the second time. The entrance of air was both seen and heard during the convulsions. |
| 31. Mussey, 1837. | Amer. Journ. Med. Sci., Feb. 1838, p. 390; Gaz. Méd., 1838, p. 394. | Subclavian vein (amputation of scapula and clavicle). | Gurgling sound. A cry of distress. Pulse imperceptible. Insensibility. Compression on vein. Usual remedies in syncope. Recovery in eight or ten minutes. | | Bubbles of air were seen entering the divided vein. |
| 32. Maugeis. | Gaz. Méd., 1838, p. 117. | Median vein (venesection.) | When about eight oz. of blood were abstracted patient uttered a feeble cry, and died instantly. | No dissection. | |
| 33. Dupuytren, 1820 (Duportail). | Gaz. des Hôp., 1837, p. 422; Gaz. Méd., 1838, p. 116. | Axilla. | No sound heard. Patient expressed uneasiness, became pale. Died in a few minutes. | Ditto. | |
| 34. Warren, 1831. | Observations on Tumours, p. 259. | Subscapular vein. | Slight venous hem: indistinct gurgling or bubbling sound. Face livid. Apoplectic breathing. Axilla compressed. Laryngotomy, artificial respiration. Death in a few minutes. | Ditto. | The vein was isolated by dissection, opened an inch from axillary vein, and its coats were not diseased. |
| 35. Goulard, 1833. | Gaz. Méd., 1833, p. 834-5. | Supposed to be axillary vein. | Very slight hem. Face convulsed. Almost immediate death. | Ditto. | |
| 36. Barlow, 1830. | Med.-Chir. Trans., 1830, vol. xvi, p. 28. | Tumour on neck; cutaneous vein. | Hissing gurgling sound. Sudden death without sigh or convulsions. | Ditto. | The sound is stated to have evidently rushed from a large divided empty vein. |
| 37. Anonymous (Amussat). | Recherches sur l'Introduction de l'Air, &c., p. 151. | External jugular vein (venesection in cholera). | No blood issued: a tube was passed into the vein towards the heart. The patient uttered a feeble sigh and died. | Ditto. | |

| CASES. | REFERENCE. | REGION OR VEIN. | SYMPTOMS AND RESULT. | DISSECTION. | OBSERVATIONS. |
|---|---|---|---|---|---|
| 38. Wattmann and Dumreicher. | Sicheres Heilverfahren, &c., p. 122. | External jugular vein. | Hissing sound. Palor. Syncope, and convulsions. Vein compressed. Symptoms ceased in half an hour after vomiting. Death on twenty-eighth day. | No dissection. | Aneurism by anastomosis in a child aged 14 months. Death was caused by pneumonia. |
| 39. Mirault. | Gueretin, Thèses de Paris, July, 1837. | Right internal jugular vein. | Whizzing sound repeated three times. Tetanic spasms. Patient revived in eight minutes. Sudden death three hours after the operation. | Ditto. | Twelve ounces of blood were not lost during the operation (extirpation of tumour of the neck). |
| 40. Clemot. | Gaz. des Hôp., 1834, No. xxiv, p. 95. | Amputation of breast. | Death some hours after operation. | Air found in veins between wound and heart, and in right cavities of heart. | |
| 41. Delpech, 1830. | Mém. des Hôp. du Midi, 1830, pp. 231-641; Gaz. Méd., 1834, p. 349. | Amputation at shoulder, *before* joint was opened. | A snuffling noise twice heard. Syncope. Death by termination of operation. | Body opened under water. An immense quantity of bubbles of air escaped on opening vessels and right cavities of heart. | A case of fungus hematodes. The vessels were greatly enlarged, but very little blood was lost. |
| 42. Roux, 1832. | Journ. des Connaiss. Médic. Chirurg. 1836; Gaz. Méd., 1837, p. 482. | Ditto. | Whizzing sound, *thought* to be heard by some. Syncope. Death by termination of operation. Symptoms came on *while deltoid muscle was being divided*, and the axillary vessels were compressed before they were divided. | Right ventricle elastic, opened under water, and eleven cubic centimeters of air collected from it. Air in coronary veins and vena cava. No air in veins of neck, axilla, or in brain. | All the air in the right ventricle could not be collected. Air on examination was found to be atmospheric. The symptoms came on while the deltoid muscle was being divided to form a flap. |
| 43. Castara, 1826. | Saucerotte, Thèses de Strasbourg, 1828. | Vein joining the subscapular vein (tumour on right shoulder). | Several rapid gurgles seeming to come from bottom of wound. Insensibility, eyes upturned, palor. Cessation of pulse and respiration. Two deep inspirations. Death without convulsions. | Right cavities of heart distended with blood, mixed with air. A little blood mixed with some bubbles of air in left cavities of heart. Blood and air in right subclavian vein and superior vena cava. No air in inferior cava. Bubbles of air in subscapular vein and veins of right arm to bend of arm, interrupting the column of blood here and there. | The body was examined twenty-four hours after death, and presented no trace of decomposition. The vein that was opened adhered to the pedicle of the tumour, on raising which the wound in the vein was seen as a round open orifice, not a line in diameter, from which bubbles of air issued on pressing the subscapular vein. |
| 44. Anonymous (Putegnat). | Thèses de Paris, 1834, No. clvi. | External jugular vein (venesection in apoplexy). | Sudden death. | Air in right auricle of heart. | |

| | | | | | |
|---|---|---|---|---|---|
| 45. Pellis, 1838. | Bull. de l'Acad., t. iii, p. 939. | External jugular vein (suicide). | Flux and reflux of blood distinctly observed in vein, which bled freely. Patient threw his head back and inspired deeply, when a gurgling noise was heard, followed by immediate death. | Heart removed: the vessels being tied, it swam on water. Air in ail the cavities, especially the right. On incising the cavities, air escaped and the heart sank. | The air in the heart was collected, analysed by M. Bischoff, and found to be atmospheric air. |
| 46. Dupuytren, 1822. | Archiv. Gén. de Méd., t. v, p. 430. | Posterior and lateral region of neck. | Sound like air entering an exhausted receiver. Patient exclaimed she was dying. General tremors. Sunk insensible and died. Cold aspersion and artificial respiration tried. | Right auricle distended with air, which escaped on opening it; contained some liquid blood. Blood mixed with air in other cavities of heart and in vessels of trunk, limbs, and brain. | The body, examined 24 hours after death, presented no trace of decomposition. The tumour was kept on the stretch and moved from side to facilitate the dissection, when the symptoms supervened. |
| 47. Beauchene, 1818. | Magendie's Journal, t. ix, p. 80. | External jugular vein, within a line of subclavian vein. | A whizzing sound, which ceased on application of finger, and returned on removing it. Patient exclaimed she was dying. Peculiar rustling sound heard in thorax. Convulsive motions. Insensibility. Death fifteen minutes after operation, which lasted half an hour. | No blood or air in any of the cavities of the heart. Numerous bubbles of air in all tbe vessels of the brain, in the vena cava inferior, iliac veins, aorta and crural arteries. Dissection eighteen hours after death. | The symptoms occurred when the clavicle, which had been divided at the centre, was pulled forward to facilitate its separation. The patient was a man, aged 23, had lost little blood, and did not appear weakened. |
| 48. Graedener. | Prov. Med. and Sur. Journ., May 14, 1842. | Tumour on the neck. | A hissing sound. Immediate death. | Cavities of heart distended by a great quantity of air. | |
| 49. Ditto. | Ditto. | Ditto. | Ditto. | Ditto. | |
| 50. Ditto. | Ditto. | Ditto. | Ditto. | Ditto. | |
| 51. Roux, 1832. | Gaz. Méd., 1833, p. 498; Journ. Hebdom., May, 1833. | Internal jugular vein. | Sound like air entering an exhausted receiver. Patient uttered a plaintive cry. Agitation. Embarrassed breathing. Apparent death. Wound compressed. Patient. A ligature round tumour. Death, *seventh* day. | Internal jugular vein divided. Cavities of heart empty. Air mixed with blood in aorta and iliac arteries. No air in veins or in vessels of brain. Lungs œdematous. Frothy fluid in bronchiæ. | The symptoms were coincident with putting the tumour on the stretch, to facilitate the dissection. |
| 52. Ulrich, 1834. | Journ. des Connais. Méd. Chirurg., t. ii, p. 91. | Ditto. | A whizzing sound, *thought* to be heard by some. Not a drop of blood flowed from vein, which remained patulous, like an artery. Soon some hem from lower end of vein. Syncope. Slight convulsions of face. Opisthotonos. Death in one minute. | Dissection fifty-two hours after death. Right auricle distended with alr, and contained no blood, Blood and no air in right ventricle. | |

| CASES. | REFERENCE. | REGION OR VEIN. | SYMPTOMS AND RESULT. | DISSECTION. | OBSERVATIONS. |
|---|---|---|---|---|---|
| 53. Gorré, 1842. | Annal. de la Chir. Fr. et Etrang., Nov. 1842, p. 305. | Ditto, near clavicle. | A gurgle from wound towards heart. Pallor. Quick respiration. Patient uttered a plaintive cry, said she was dying, and died within a minute. An attempt was made to close the orifice of the vein and compress the thorax. | Much frothy liquid blood in right cavities of heart and internal jugular vein. Some bubbles of air in superior cava, subclavian, axillary, brachial veins; veins of brain. In aorta and iliac arteries. | The hemorrhage was trifling. The body was examined 24 hours after death. The orifice in the vein was oblong and gaping. The symptoms occurred when the tumour was put on the stretch. No air or blood in right heart. |
| 54. Handyside, 1837. | Edinb. Med. and Surg. Journal, 1834, vol. ix, p. 209. | Left facial vein; right external and internal jugular veins (suicide). | | Air in both internal jugular and thyroid veins, both venæ cavæ, veins of liver, spleen, and kidneys. In left carotid aorta, femoral, popliteal, brachial arteries. Some air and no blood in all the cavities of the heart. | Dissection 26 hours after death in very dry cold weather. Left spinal accessory nerve and right pneumo-gastric nerve divided. A pound and a half of blood had been lost. |
| 55. Verrier, 1806. | Bull. de l'Acad., 1837, p. 364. | External jugular vein of horse (venesection). | Loud gurgling, followed by usual serious symptoms on ceasing compression below to terminate operation. Recovery. | | |
| 56. Bouley, 1821. | Magendie's Journ., t. i, p. 197. | Ditto. | On removing pressure below puncture horse fell as if thunderstruck. Blood allowed to flow again. As it issued horse gradually revived and recovered. | | |
| 57. Gerard. | Gaz. Méd., 1838, p. 117. | Ditto. | Ditto. | | |
| 58. Bouley, 1838. | Bull. de l'Acad., 1838, t. iii, p. 465. | Ditto (left side). | While securing puncture, hurried laboured breathing. Pulse ceased. Tremors. Death in a few minutes. | Frothy blood in left jugular vein. Air mixed with coagulated blood and some liquid blood in right cavities of heart. Frothy blood in pulmonary artery and its minutest branches. Clot with globules of air in left heart. Air in veins of abdomen and brain. | M. Bouley was absent when the air is supposed to have entered the vein. The groom did not perceive any sound. One lung was hepatized. |
| 59. Ditto, 1841. | Bull. de l'Acad., 1841, t. vi, pp. 178-83. | Ditto. | Embarrassed breathing. Pulse imperceptible. Blood, mixed with numerous bubbles of air, flowed from vein. Death in seven hours. | Frothy blood in right cavities of heart, pulmonary artery, and right cavities of heart, in veins of abdomen and brain. | Ditto. Dissection two hours after death. |
| 60-61. Ferries. | Ditto, p. 183. | Ditto. | Death soon after the operation. | | No further particulars given. |
| 62 to 67. Leblanc. | Gaz. Méd., 1838, p. 117. | Ditto. | | | No particulars given. Not stated whether any of the animals died. It is merely said that M. Leblanc saw air enter the vein during venesection in six horses. |

It would lead us to tedious and unnecessary prolixity were we to minutely analyse each of the cases in the foregoing table, but we must consider the value of the objections made to some of them at least. To begin with the simplest case, Velpeau disposes of the 9th, 10th, 22d, and 40th, by alleging that the veins opened were beyond the limits of danger; but not only is this an assumption without any proof, but it is certainly erroneous, at least as regards the 10th, 22d, and 41st cases, for the axilla (case 22) is within the limits of danger, and as in the 41st case, air was found in the veins between the wound and the heart, and in the right cavities of the heart, it is proved that the veins divided during the amputation of a breast may admit air. Velpeau admits that air may have entered the veins in the 14th, 15th, and 18th cases, but if so, he says, we can only conclude from them what is already known from experiments on animals, that the accident is by no means uniformly fatal. We, of course, readily admit this truism, but confess we cannot understand how it militates against the fatality of the accident in other cases; unless, indeed, the fact that some animals survived entrance of air into the veins is also held to prove that none of the animals submitted to experiment died from this cause. The 37th and 44th cases are objected to, because the veins were too small and remote from the heart, and because death was sudden and without convulsions; the former objection we have already seen is unfounded, and in the 44th case the relations of the vein to the tumour were precisely those which it is generally admitted may extend the sphere of danger beyond its ordinary limits, and to veins smaller than usual. As to the second objection, convulsions by no means uniformly occur in animals, and independently of the fact that the rapidity of death varies in different animals, M. Segallas's experiment shows that death may be immediate even in dogs. The same objections are advanced against the 42d, 43d, 47th, 48th, and 53d cases, with the addition that, as regards the 42d case, the sound of air entering a vein in animals is not a *reniflement;* that in the 42d and 43d cases we do not know what was the condition of the blood in the right side of the heart; that in the 47th and 53d cases, the air in the heart was not mixed with the blood; and that in the 48th case air was found in numerous vessels, but not in the heart. We have already seen how much the sound of air entering a vein varies in many respects, and when we further consider that its tone must vary with the size and tension of the edges of the aperture through which air passes, we might almost call such an objection mere cavilling. Independently of theoretical considerations, the influence of the condition of the orifice of the vein on the sound is well exemplified by one of Dr. Wattman's cases (26th), in which the sound ceased when the lips of the orifice were sufficiently separated. Though it is digressing a little, it is convenient to notice here M. Blandin's objection, that all the cases in which a "hissing" sound was heard are probably a mistake, as he was once alarmed by this sound, but found it caused by the rapid projection of blood from an artery against the adjacent tissues (Bull. de l'Acad. 1837, p. 903); and also his allegation, that he has repeatedly known a deceptive *lapement* and *gargouillement* to have been heard during an operation, whence he argues that no conclusion can be deduced from the mere occurrence of those sounds. (Des Accidents qui peuvent survenir pendant les opérations.) But those objections, be they ever so well founded, can only apply to such cases as the 12th, 13th, 14th, 15th, and 30th, in which a sound resembling that of air entering a vein was the solitary symptom observed. But to return to the cases specified above as objected to, because of the condition of the air in the heart or the absence of air from that organ, it has been already seen that the objections are of no weight; the air was sometimes found unmixed with blood, even in the very set of experiments on animals which are arbitrarily assumed by M. Velpeau as a standard of comparison, and Poiseuille has found the heart completely empty of air. We have, we believe, answered all the objections which have been made to any of the cases above adverted to (or indeed to any case worthy of serious examination), with the exception of one, viz. that there is no evidence that a volume of air, sufficiently large to cause either death or the formidable symptoms frequently observed, entered the veins in any of the recorded cases, and that from the rapidity of the supervention of those symptoms or even of death, it is very unlikely that such a volume of air could have entered; and the following computation made by M. Barthélemy has been appealed to in support of this objection. M. Barthélemy found that on an average *four* litres of air quickly (within 7 or 8 seconds) injected into the jugular vein of a middle-sized horse, i. e. weighing about 450 kilogrammes, caused death in a few minutes; and if we suppose (which M. Barthélemy, however, by no means assumes) that the same relation exists between the weight of different animals and the quantity of air necessary to kill them, he computes that it would, on that hypothesis, require the rapid introduction of a little more than $\frac{3}{5}$ of a litre of air into the vein of a middle-sized man to cause death. (Bull. de l'Acad. t. i, p. 370-5). With respect to this hypothetical calculation, it is sufficient to say that its basis utterly fails, for M. Renault found that the sudden injection of a *litre and a half* of air always sufficed to kill a horse. (Bull. de l'Acad. 1841, p. 181.) We might further speculate that the higher organization of man, his upright posture, the shortness of his neck, the greater proximity to the heart of the vessels opened in almost all the recorded cases, than of those operated on in most of the experiments on animals, may favour the deleterious action of air admitted into the human veins, but without dwelling on such inconclusive speculations, we really think it sufficient to read the account of the appearances on dissection in some of the fatal cases, especially the 42d, 43d, 46th, 47th, 48th, and 54th, to see that a very large quantity of air did enter the circulating system in those cases, fully as much, even relatively to the size of the animals, as is described to have been found in the dogs experimented on by the committee of the French Academy of Medicine. This large quantity of air was also admitted with great rapidity, whence its influence was proportionally greater, and if its presence is not admitted to be sufficient to explain death in those cases, we think it impossible to avoid the conclusion that neither can the death of the animals, submitted to experiment by the committee so often referred to, be attributed to the entrance of air into their veins.

If, instead of merely answering objections, we examine the circumstances of some of the foregoing cases, it seems really difficult to understand how it can be denied that death resulted from entrance of air into the veins, unless it is at the same time contended that all the experiments on animals respecting the spontaneous entrance of air into the veins, are also fallacious and insufficient to establish the reality of death from this cause. In Beauchene's case for example, the external jugular vein is opened within a line of the subclavian vein (almost equivalent to a wound of the latter vein itself): a whizzing sound is heard, which ceases on applying the finger to the wound, and returns on its removal; the patient exclaims she is dying, *a peculiar rustling sound* is heard *in the thorax,* convulsive motions with insensibility supervene, and death occurs in 15 or 20 minutes; on dissection numerous bubbles of air are found in the vessels of the head, trunk, and lower extremities: the chain of evidence is complete, and yet this case is rejected because of the erroneous assumption that the wound was beyond the limits of danger, and the equally unfounded objection that the heart uniformly contains air after death from admission of air into the veins. The 44th case is as strong, stronger we think it could not be, and what can be more conclusive than the 54th case. But to examine these cases, and some of the others further, would in effect be only repeating in another form what has been already said.

We have left unnoticed many cases in the table, but we willingly admit that we have not sufficient particulars respecting several of them, to form any opinion about them one way or the other. And as our object is merely the general one of endeavouring to establish the fact of death occurring from the spontaneous admission of air into the veins, a particular examination of all the cases, especially of the more doubtful ones, would be quite beside our purpose.

Even those who question that death has ever resulted from admission of air into the veins during an operation, admit that it is prudent in practice to take due precautions for its prevention, and to be prepared to remedy its consequences should it occur; and as some of the remedial measures proposed, are based on the views taken of the action exerted by air when admitted into the vascular system, we must advert to those views.

Brunner, Camerarius, Harderus, Sprœngel, Nysten, Magendie, Dupuytren, Amussat, and others, attributed death to interruption of the circulation from extreme distension of the right cavities of the heart; Harderus, and subsequently Nysten, conceived that the over-distension of the fibres of the heart destroyed their contractility, and Magendie is of opinion that the great distension of that organ is caused by the sudden expansion of the air, from its temperature being elevated on entering the vessels. M. Denot is also of opinion that death arises from the circulation being suspended by the presence of air in the heart, but he does not think that this occurs because the heart is overdistended, but because the tricuspid valves, which permit some reflux even of the blood, easily allow the reflux of an elastic fluid, and the air, therefore, plays to and fro between the right cavities, which thus contain a fluid which they are unable to propel; the access of venous blood to the lungs is, he says, thus interrupted and death results as if the pulmonary artery were tied. (Gaz. Méd. 1837, p. 726.) Mr. Wing also refers death to disturbance of the circulation; the distension of the right cavities of the heart causes the valves to act imperfectly, and the blood regurgitates at each contraction of the ventricle, this reflux increases the disturbance of the heart's action, which increases till the action of the organ ceases, not gradually, but suddenly. (Boston Med. and Surg. Journ. and Gaz. des Hôpitaux, 1835, p. 108.) Arrest of the circulation is likewise the chief cause of death admitted by M. Mercier, who thinks that there is—1st, venous stasis, consequent on accumulation of blood in the right auricle; and, 2dly, that the action of the right ventricle condenses the air in the pulmonary artery, which causes the blood to be imperfectly propelled through the venous capillaries, the more so as it experiences great difficulty in traversing them in consequence of being frothy from admixture with air; the blood, therefore, reaches the left heart, and the brain very slowly, and their functions are proportionally disturbed, especially, as whatever blood does reach them is mixed with air. (Gaz. Méd. 1837, p. 481.) M. Poiseuille adopts a portion of M. Mercier's theory; he considers that the *sole* and *only* cause of death is complete cessation of the pulmonary circulation from the frothy blood being unable to traverse the capillaries of the pulmonary artery, the lung is, therefore, obstructed, and little or no blood gains the pulmonary veins. Distension of the right cavities of the heart, whether with air or with blood, or both, is, he thinks, simply the result of obstruction of the pulmonary circulation. (Gaz. Méd. 1837, p. 671.) M. Gerdy's opinion is very similar, but he refers the obstruction of the pulmonary circulation to the mere presence of air in the pulmonary artery, without reference to the frothiness of the blood impeding its passage through the pulmonary capiliaries (Bull. de l'Acad t. ii, p. 287); a view which is also adopted by M. Segallas. (Op. cit. p. 425.) M. Bouillaud adopts a mixed opinion, and considers that the principal causes of death are—enormous distension of the right cavities of the heart, produced by the air which is expanded by the heat of the blood;—the difficulty experienced by the frothy blood in traversing the pulmonary capillaries,—possibly the compression exerted by the air in the pulmonary system, obstructing respiration and producing asphyxia;—and probably pressure on the brain in the cases where the air reaches the vessels of that organ.

M. Piedagnel (Magendie's Journ. 1829, t. ix, p. 6) and M. Leroy (Archiv. gén. de Méd. 1823, t. iii, p. 142) think that dilatation of the air from its sudden elevation of temperature, ruptures the capillaries of the lungs, which thus become generally emphysematous, and the circulation at once stops.

Bohn long since considered that air injected into the veins acted as a powerful and rapid poison. M. Marchal de Calvi has recently received this theory, and maintains that the poison is carbonic acid; for that gas must be evolved when atmospheric air encounters venous blood, and the bright red colour of the venous blood in animals killed by throwing air into the veins proves that the blood is decarbonized. He thinks that the more rapidly fatal effects of air expired from the lungs favour this view; but he acknowledges that Nysten's experiments on injecting carbonic acid gas into the veins discountenances his theory. We should rather say they completely disprove it. (Annales de Chirurg. Franc. et Etrang. Nov. 1842, p. 302.) Bichat's theory that air killed by irritating the brain, perhaps comes under this category; but its erroneousness is shown, as Nysten observed, by the fact that the air by no means constantly reaches the brain. Nysten, however, thought that when air did penetrate to the vessels of the brain, it acted injuriously by compressing that organ.

The opinion of MM. Piedagnel and Leroy now finds no countenance. No emphysema of the lungs existed in any of the experiments of the commission. Mr. Cormack blew air with much force into the veins, and no emphysema resulted, and he showed that the natural appearance of the rabbit's lung, which much resembles a human emphysematous lung, probably misled M. Piedagnel.

The experiments of M. Barthélemy, coupled with some facts observed by many others, disprove, we think, all the theories which refer death to suspension of the circulation, whether caused by distension of the heart, viscidity of the frothy blood, or any other cause. M. Barthélemy found that the sudden injection of four litres of water, a fluid perfectly miscible with blood, produced the same effects as the injection of the same quantity of air. As to distension of the heart, that organ is by no means always distended, its volume and consistence are often perfectly natural, especially it would seem in the horse, and the air instead of being accumulated in it, is disseminated through the circulating system even when the horse has lived but a few minutes. Sometimes on opening dogs, rapidly killed by the entrance of air into the veins, the heart is found contracting, and M. Barthélemy found the heart contract in horses till the last expiration. M. Barthélemy also performed the following experiment. He cut off the tail of a horse, and the blood jetted from the coccygeal arteries; he then threw four litres of air into the jugular vein, the jet from the arteries then intermitted, but soon became larger and stronger than previous to the intermission; after three or four minutes the flow of blood became feeble, but continued uninterruptedly till the animal expired, so that the circulation and the respiration ceased almost simultaneously. M. Barthélemy performed this experiment on ten horses; in one animal the arteries jetted after respiration ceased, and in another they continued to bleed for one minute after the last expiration. (Bull. de l'Acad. t. i, pp. 377-61.)

We cannot then consider any of the foregoing explanations satisfactory, neither does the following view, proposed by Dr. Wattmann, seem more so. The ordinary condition of fulness of the vascular system allows the heart a moment's rest after each contraction; but the admission of air renders the vascular system full to tension, and then neither the fibres of the heart that contract, nor those that dilate the organ, are allowed the necessary alternation of rest and exertion; thence the activity of the heart and its influence on the circulation diminish every moment, and at the same time the blood reaching the lungs mixed with air and in diminished quantity is imperfectly decarbonised, and its electro-magnetic conducting power correspondingly diminished. Consequently, whatever portion of blood does reach the several organs through the systemic circulation, is incapable of stimulating them properly, whence their functions become suspended.

A variety of precautions have been recommended to prevent the entrance of air into the veins during an operation, in a region where that accident may be apprehended. Experiments on animals having shown that there is more danger in the vertical position, M. Lisfranc and others recommend that the patient should be placed in the horizontal posture. Amussat and others consider that the most important and effectual precaution is to permanently compress the veins between the part operated on and the heart, but this would often be impracticable. Dupuytren attached particular importance to avoiding putting the parts on the stretch while the dissection was being performed, and, to facilitate this, recommended, in the extirpation of a tumour, that it should be removed piecemeal if necessary. M. Velpeau advises that motions of the neck or arm should be avoided, and that the deep attachments of a tumour situate near a large vein should be firmly grasped or encircled by a ligature before they are divided. Cormack recommends that the patient should be made to inspire before a vein is divided; and M. Gerdy, with the view of limiting the motions of inspiration, considers that the thorax should be encircled with a tight bandage. (Bull. de l'Acad. t. i, p. 909.) Of those recommendations the last only is objectionable; but the others, however useful, cannot always be adopted, and it must, we think, be admitted that no care, no precaution, however great, can afford any certain guarantee against the occurrence of the accident.

When symptoms indicating the admission of air into the veins occur, a variety of means have been proposed to remedy the consequences, and many of those means are founded either on the results of experiments on animals, or the view taken of the mode in which the air acts injuriously.

Nysten concluded, from his experiments on dogs, that sudden compression of the abdomen and thorax was beneficial in remedying the symptoms in question. M. Amussat is of opinion that the most effectual remedy we possess, is to repeatedly compress the thorax during expiration only; the air he thinks may be thus expelled from the heart, and he recommends that the vein should at the same time be immediately closed, provided a small quantity only of air has entered; but if much air has gained admission, and especially if frothy blood issues from the wound, closing the vein he thinks hastens death, while by leaving it open, at least during expiration, and while the thorax and abdomen are compressed, the left cavities of the heart may be emptied of air and life preserved. M. Mayor also recommends repeated pressure on the thorax, which he considers useful, by exciting artificial respiration as well as by expelling the air from the heart, and recommends the orifice in the vein to be always closed. (Bull. de l'Acad. t. iii, p. 938.) MM. Gerdy and Velpeau object to compression of the thorax, that even if it is useful when applied to the yielding thorax of the dog, the human thorax could not be safely compressed so as to act on the heart. M. Blandin also objects that the valves of the veins would prevent the reflux of air; but to this M. Amussat replies that the veins are destitute of valves at the lower part of the neck, as is shown by the flux and reflux of the blood. (Bull. de l'Acad. t. i, pp. 899-912; t. ii, pp. 20-275).

As the presence of air in the circulating system is the cause of the mischief, it seemed rational to endeavour to remove it, more especially as its chief injurious action was considered to consist in distending the heart, and as in some experiments the animals revived when frothy blood escaped from the wounded vein. The proceedings above adverted to contemplated the expulsion of the air from the heart, but Magendie conceived the idea of removing it directly by aspiring it through a tube passed into the vein towards the heart. Mr. Cormack in his experiments found this proceeding sometimes succeed, and sometimes fail, but thought any good effects derived from it were more attributable to the evacuation of blood than of air. Mr. Cormack, however, it is right to add, notices the danger of air entering the tube, and though MM. Blandin, Rouchoux, Segallas, and some others (Bull. de l'Acad. t. i, pp. 901-3; t. ii, p. 428), theoretically recommend this practice, no one, we believe, has had the hardihood to apply it to the human subject. Even were it safe, there would rarely be time for so delicate a proceeding; in most cases it would be absolutely impracticable, and if practicable it would, to use M. Barthélemy's words, but open the door to the enemy.

Bloodletting has been found useful in experiments on animals by Mr. Cormack, and by MM. Bouley, and Leblanc. More recently it has been strongly recommended by M. Marchal de Calvi. (Annales de Chirurg. Franc. et Etrang. Nov. 1842, p. 304.) This practice certainly deserves further experimental investigation.

Though bloodletting is recommended on the grounds that it diminishes the contents of the circulating system, and thus relieves the over-distension of the heart and vessels, yet M. Mercier advises a proceeding which, so far as it is efficient, must have a contrary effect; he recommends that the abdominal aorta and axillary arteries shall be compressed, with the view of directing towards the brain whatever blood does reach the arterial system, and states that he found this method very beneficial in experiments on the dog. (Gaz. Méd. 1838, p. 236.)

M. Poiseuille, from his theory as to the mode in which death occurs, recommends that the patient shall be placed in such a position that the air in the right side of the heart shall not correspond to the orifice of the pulmonary artery. (Gaz. Méd. 1837, p. 671.) M. Denot, on the contrary, influenced by his theory, advises that the patient shall be placed in the dextro-dorsal posture, to favour the flow of blood from the right ventricle into the pulmonary artery. (Gaz. Méd. 1837, p. 728.)

When air is supposed to have entered a vein, there is no indication so obvious as to prevent its further access by immediately compressing the wound, and accordingly reference to the Table of cases will show how generally that practice has been adopted. M. Velpeau, it is true, doubts how far closing the orifice of the vein is dangerous or the reverse, for though so doing prevents air entering, it equally prevents its exit. We have also seen the limitations M. Amussat puts to this practice; but both those gentlemen adopted it in their own cases. (Cases 10 and 28.) But, with those partial exceptions, we may fairly say this practice is generally approved of, and has been generally adopted. Now this is precisely the practice which Dr. Wattmann has written a book to recommend as "a certain remedy," and which he inculcates in the two following paragraphs with whatever emphasis is conveyed by printing in capital letters.

"The required therapeutic Indication is to prevent as quickly as possible, and permanently, the Entrance of air into the Veins."

"The indication can only be fulfilled by the Instantaneous and permanent closure of the wound of the vein." (p. 94.)

As we have not the slightest doubt of the propriety of this practice, we shall not occupy space by detailing the reasons adduced by Dr. Wattmann in its support. We need only say that as regards the permanent closure of the orifice in the vein, Dr. Wattmann is satisfied with compression whenever the wound can be completely closed, or when a flap or one of the lips of the wound can be laid over the vein and retained so by sufficient compression. When this cannot be effected, he either includes the vein in a ligature (as in the 20th and 21st cases, in which the axillary vein was tied), or if there is but a small opening in a large trunk, he pinches up the orifice in a forceps and ties a ligature round the portion of the coats of the vessel included in the instrument; in this way he successfully secured a wound of the internal jugular vein in the 27th case. Should the wounded vein lie too deep to thus apply a ligature, as in the case of the subclavian vein in a fat person, Dr. Wattmann proposes that the lips of the wound should be pinched up in a small light spring forceps, which should be left *in situ* as long as might be necessary. This mode of securing a partially wounded vein is by no means so original as Dr. Wattmann seems to imagine; for Mr. Guthrie, in a case of suicidal cut throat, included a small wound in the internal jugular vein in a circular ligature. (On the Diseases and Injuries of Arteries, p. 328.) We do not pretend to deny that the ligature of a large vein may not sometimes be necessary, but we believe it will be rarely so; we have seen the axillary vein wounded during the extirpation of schirrous glands in the axilla, a dossil of lint was laid on the wound, and all went well. In the Table of cases above given, the internal jugular vein was wounded in the 29th, 30th, and 31st cases, and no ligature was requisite, simple compression answered every purpose.

Dr. Wattmann is of course perfectly aware that closing the orifice in the vein has often been practised; but he says it has only been considered of secondary importance, or at most as a method to be employed in conjunction with others, and not as the means on which our sole and only dependence should be placed. He admits, however, that if a sufficient quantity of air has gained admission to produce general symptoms, especially syncope, that the means usually had recourse to under such circumstances should not be neglected, such as the horizontal posture, free access of air, cold aspersion, the application of the vapour of ammonia or of strong vinegar to the nostrils, warmth to the extremities, &c. Artificial respiration he deems useless.

On reviewing the various modes of treatment which have been recommended to remedy the effects of admission of air into the veins, it must, we think, be admitted that our resources are very limited, and perhaps the only really useful measures are closing the aperture in the vein, and the general means alluded to in the last paragraph. We should, however, be inclined also to practise moderate compression of the thorax during expiration, not on the grounds recommended by M. Amussat, but in accordance with M. Mayor's view of exciting artificial respiration. Dr. Warren in one case performed laryngotomy, in order to employ artificial respiration, but this we consider useless and injurious as involving loss of time; injecting a saline solution into the veins, proposed by the same gentleman, is still more to be deprecated. Bloodletting would appear from some experiments on animals to be useful, but further experiments are required before it can be recommended with any confidence.

We shall not examine Dr. Wattmann's speculations respecting the medico-legal import of entrance of air into the veins; for he makes several assumptions which we have shown to be erroneous. Thus he says, that in every examination after death from entrance of air into the veins, the right cavities of the heart have been found to contain air mixed into a froth with blood, and air has also been found in the vessels of the brain; and he also admits that the presence in the heart of free air unmixed with blood, indicates that the air has been admitted after death. We shall, therefore, here terminate our notice of Dr. Wattmann's work, which, though scarcely perhaps in all respects what we might have anticipated from the high attainments and eminent position of the author, is certainly a work of much research, and taken altogether is, we believe we may safely say, certainly the best extant on the subject of which it treats. As that subject is one respecting which opinions are very unsettled, and which is not very likely to come frequently before us, we have taken the present opportunity to consider it somewhat in detail.

---

